# The Association Between Breast Arterial Calcification and Subsequent Coronary Artery Calcification: A Systematic Review

**DOI:** 10.7759/cureus.93339

**Published:** 2025-09-27

**Authors:** Stephanie Nagy, Christopher Rodriguez, Adriana Gurreri, Kitty Daniel, Christos G Mihos, Marc M Kesselman

**Affiliations:** 1 Rheumatology, Nova Southeastern University Dr. Kiran C. Patel College of Osteopathic Medicine, Davie, USA; 2 Radiology, Mount Sinai Medical Center, Miami, USA; 3 Cardiology, Mount Sinai Heart Institute, Columbia University College of Physicians and Surgeons, Miami Beach, USA

**Keywords:** atherosclerosis, bac, breast artery calcification, breast artery calcium scoring, cad, cardiovascular disease, coronary artery calcium scoring, coronary artery disease, ct angiogram

## Abstract

Cardiovascular disease (CVD) remains the leading cause of mortality globally. While coronary artery calcium (CAC) scoring is the current gold standard for detecting subclinical atherosclerosis, recent research has highlighted breast arterial calcification (BAC), often incidentally identified on screening mammograms, as a potential non-invasive marker of CVD risk. BAC has been significantly associated with an increased risk of adverse cardiovascular outcomes, including ischemic and hemorrhagic stroke, peripheral vascular disease, and heart failure. This systematic review comprises 14 studies aimed at better understanding the association between the presence of BAC and CAC. The studies consisted of 5,249 women who underwent both BAC and CAC scoring, primarily post-menopausal, with an average age of 57.8 years. The time interval between BAC and CAC assessment ranged from same-day imaging to 2.94 years (mean: 16.2 months; median: 12 months). Across all studies, a consistent positive correlation was observed between the presence of BAC and CAC. Notably, women with both BAC and CAC tended to be older and exhibited a higher burden of coronary artery stenosis, an increased number of affected vessels, and more extensive calcification distribution. The pooled sensitivity, specificity, positive predictive value, and negative predictive value of BAC for detecting CAC were 38.9%, 88%, 73%, and 57.2%, respectively. These findings suggest that the presence of BAC may serve as a useful tool for supporting the presence of CAC in at-risk patients. Given its accessibility, cost-effectiveness, and high specificity, BAC holds promise as a screening adjunct for early CVD detection in women. Standardized guidelines are needed to support radiologists in the consistent reporting of BAC on mammograms and to inform primary care providers on appropriate follow-up and cardiovascular screening for patients with positive BAC findings.

## Introduction and background

Epidemiology of breast arterial calcification

Cardiovascular disease (CVD) is considered to be the most common cause of death worldwide, a trend that has been consistent since the 1920s in the United States [1]. The disease accounted for 941,652 deaths in the US in 2022 alone, and worldwide, it accounted for 19.8 million deaths [2,3]. CVD is an all-encompassing term that describes any damage to the heart and/or blood vessels, including conditions of myocardial infarction, stroke, and coronary artery disease (CAD), among others. The most prevalent etiology of CVD is coronary artery calcification (CAC) and atherosclerosis, and this plaque-induced narrowing of arteries exists in close physiologic interplay with hypertension and hyperlipidemia [4]. CAC is a result of calcium deposits on coronary artery walls, which, over time, can progress and cause narrowing, commonly known as atherosclerosis [5]. CAC scoring is a valuable tool for CVD risk stratification and guiding clinical management, particularly in individuals with intermediate to high risk who are asymptomatic.

An increasingly recognized comorbidity associated with CVD in women is breast arterial calcification (BAC). Calcifications within the breast tissue are usually found incidentally during a mammogram. While many are benign, certain patterns may indicate a precancerous condition and require further workup. Benign BAC, consisting of calcium oxalate or calcium phosphate, can appear on imaging as two dense, linear, parallel calcifications referred to as a “tram-track” appearance that is usually oriented away from the breast ducts [6,7]. They arise in the medial layer of breast arteries as part of a condition known as Mönckeberg medial calcific stenosis and can be related to the future incidence of CVD. The prevalence of BACs is approximately 27% and often co-exists in the setting of pro-inflammatory cardiovascular comorbidities such as diabetes mellitus, hypertension, and metabolic syndrome [8-10]. Importantly, the presence of BACs has been associated with a nearly two-fold increased risk of cardiovascular death, ischemic stroke, and heart failure, respectively [11].

Proposed mechanisms

The pathophysiology of BAC development is currently being analyzed, with the leading hypothesis focusing on vascular smooth muscle cells. These cells can undergo a phenotypic switch to osteoblast-like cells that secrete calcium salts and matrix vesicles, leading to the formation of hydroxyapatite crystals that facilitate mineralization within the vascular walls [12]. This phenotypic switch can be spontaneous or secondary to multiple inflammatory processes, using the extracellular signal-regulated kinase (ERK) pathway in both cases. Spontaneously, tissue growth factor (TGF) stimulates ERK to activate the transcription factor Runx2, which is known to be essential in the osteogenic process. Runx2 controls the transdifferentiation of vascular smooth muscle cells, leading to the development of calcification [13]. When it comes to inflammation, pro-inflammatory cytokine interleukin (IL)-18 and the ERK pathway can activate a TRMP7 cation. TRMP7 is necessary to maintain magnesium levels in the body, which regulates vascular calcification and prevents the osteogenic differentiation of vascular smooth muscle cells, as well as the expression of calcification inhibitors of osteopontin and matrix Gla protein [14]. Dysregulation of the TRMP7 channel results in uncontrolled calcification of the vascular wall and activation of the Runx2 pathway. Other pro-inflammatory cytokines such as IL-6 and tumor necrosis factor (TNF)-alpha can also cause a build-up of reactive oxygen species, which have also been shown to activate the ERK pathway and Runx2 [15]. 

Rationale for review

The development of breast and coronary calcifications has similarities in risk factors such as aging, hormonal influences, post-menopausal status, and metabolic syndromes [16,17]. These similarities, plus the added risk factors, prompt a greater exploration of the relationship between BAC and CAC. Given the widespread and guideline-directed use of routine mammography for the screening and follow-up of breast cancer patients, the potential utility of BAC presence to identify patients at risk for CAC and CVD is compelling. The present systematic review will analyze the published data on the association between BAC and CAC as an indicator of CAD risk in women. 

## Review

Methods

*Databases and Search Terms*

A systematic literature review was performed using OVID (MEDLINE), EBSCO (CINAHL Complete), EMBASE, and Web of Science using the search terms “breast artery calcification or breast arterial calcification or mammary artery calcification or coronary artery calcium score or coronary artery calcinosis or coronary calcium score or coronary calcium or coronary artery disease or CAD.” To ensure the recency of the articles and an updated review on this association, only articles published between 2015 and 2025 were assessed. The articles were analyzed in a step-wise process by first evaluating the title and abstract for relevance and then assessing the full-text manuscript. The Nova Southeastern University library database was used to access databases and full-text articles.

S*election Criteria*

**Figure 1:**
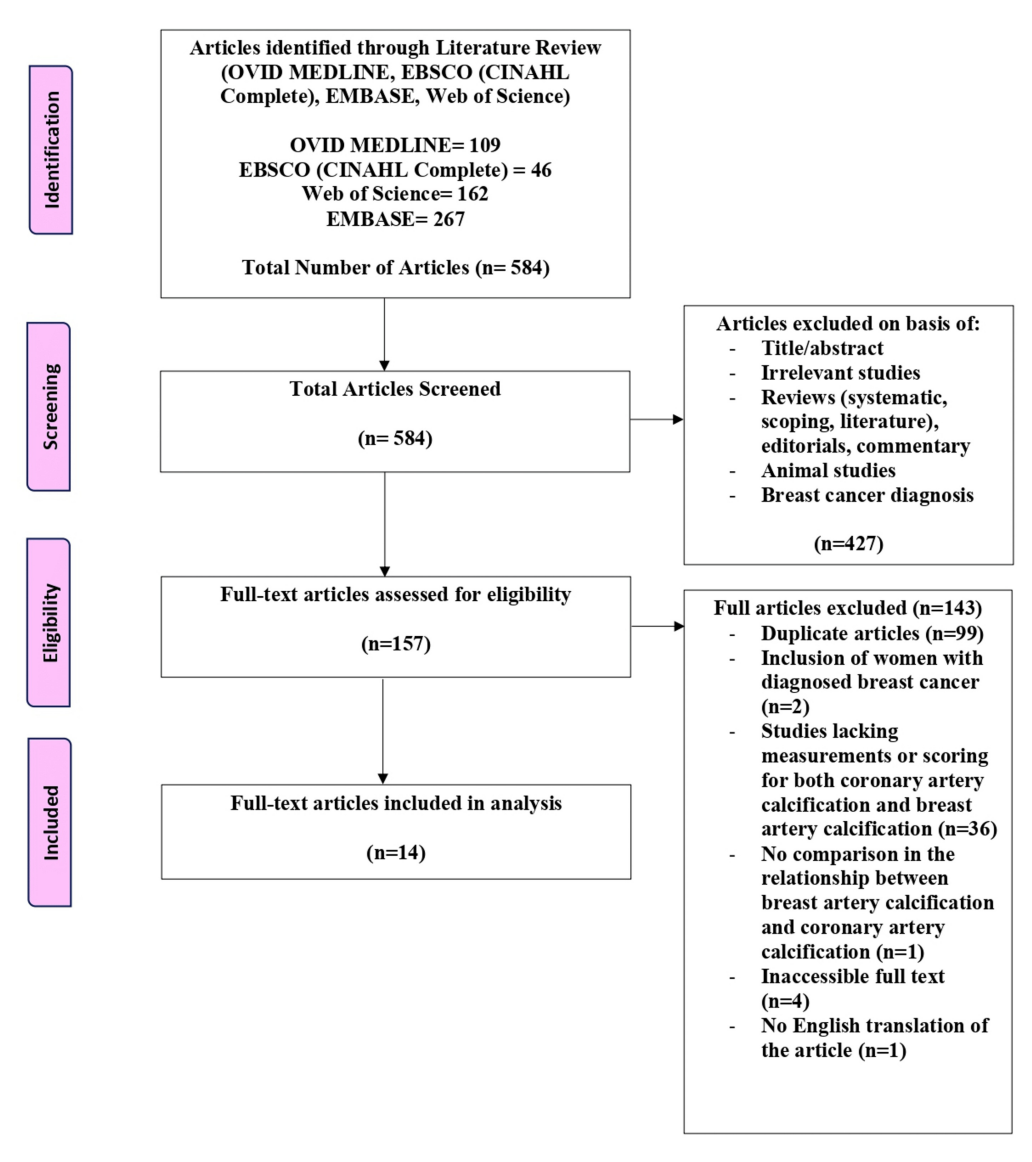
Preferred Reporting Items for Systematic reviews and Meta-Analysis

For this review, we included randomized controlled trials, cross-sectional studies, observational studies, and cohort prospective and retrospective studies. Abstracts were included if they met all of the inclusion criteria. The study population included women who underwent both a mammogram and CT angiography to identify BAC and CAC, respectively. The primary outcome was the observed association between BAC and CAC. Articles in a non-English language were included, but an English-translated version should be available. Studies excluded from this review were literature, systematic or scoping reviews, animal studies, editorials, and in vitro-focused outcomes. Articles were excluded if the patient did not undergo imaging for both BAC and CAC scoring, analysis of the relationship between the calcification was not assessed, and if women were included with diagnosed breast cancer due to malignancies also presenting with breast calcifications in some cases. Two reviewers completed a blinded review process of the articles to decide on their inclusion or exclusion based on the determined criteria, and a third reviewer was used to break any ties. The Preferred Reporting Items for Systematic reviews and Meta-Analyses (PRISMA) guidelines were followed and used to develop a flow diagram of the selection criteria for reproducibility (Figure 1) [18].

Critical Appraisal 

The studies were analyzed using the Joanna Briggs Institute critical appraisal tool (Table 1). 

**Table 1:** Joanna Briggs Institute critical appraisal tool

Article	Were the two groups similar and recruited from the same population?	Were the exposures measured similarly to assign people to both the exposed and unexposed groups?	Was the exposure measured in a valid and reliable way?	Were confounding factors identified?	Were strategies to deal with confounding factors stated?	Were the groups/participants free of the outcome at the start of the study (or at the moment of exposure)?	Were the outcomes measured in a valid and reliable way?	Was the follow-up time reported and sufficient to be long enough for outcomes to occur?	Was the follow-up complete, and if not, were the reasons for loss to follow-up described and explored?	Were strategies to address incomplete follow-up used?	Was an appropriate statistical analysis used?
Chadashvili et al. [19]	Yes	Yes	Yes	Yes	Yes	Yes	Yes	Yes	Yes	N/A	Yes
Yurdaışık et al. [20]	Yes	Yes	Yes	Yes	Yes	Yes	Yes	Yes	Yes	N/A	Yes
Kamel et al. [21]	Yes	Yes	Yes	Yes	Yes	Yes	Yes	Yes	Yes	N/A	Yes
Yoon et al. [22]	Yes	Yes	Yes	Yes	Yes	Yes	Yes	Yes	Yes	N/A	Yes
Margolies et al. [23]	Yes	Yes	Yes	Yes	Yes	Yes	Yes	Yes	Yes	N/A	Yes
Fathala et al. [24]	Yes	Yes	Yes	Yes	Yes	Yes	Yes	Yes	Yes	N/A	Yes
Yoon et al. [25]	Yes	Yes	Yes	Yes	Yes	Yes	Yes	Yes	Yes	N/A	Yes
Deeg et al. [26]	Yes	Yes	Yes	Yes	Yes	Yes	Yes	Yes	Yes	N/A	Yes
McLenachan et al. [27]	Yes	Yes	Yes	Yes	Yes	Yes	Yes	Yes	Yes	N/A	Yes
Solyu et al. [28]	Yes	Yes	Yes	Yes	Yes	Yes	Yes	Yes	Yes	N/A	Yes
Oksul et al. [29]	Yes	Yes	Yes	Yes	Yes	Yes	Yes	Yes	Yes	N/A	Yes
Saenger et al. [30]	Yes	Yes	Yes	Yes	Yes	Yes	Yes	Yes	Yes	N/A	Yes
Seifi et al. [31]	Yes	Yes	Yes	Yes	Yes	Yes	Yes	Yes	Yes	N/A	Yes
Bui et al. [32]	Yes	Yes	Yes	Yes	Yes	Yes	Yes	Yes	Yes	N/A	Yes

Results

After completing the article selection, 14 articles were included in this paper, analyzing 5249 patients, 1241 with BAC and 4008 without BAC. All patients included in the study were women; the majority were post-menopausal, with an average age of 57.8 years. Analysis of ethnicity could not be completed due to studies lacking the reported data.

Each study included was analyzed for the number of patients, mean age, BAC score, correlation of BAC with CAC, the analysis of BAC score to CAC scoring based on the Agatston scoring system as that is the widely and most established system in place for CAC scoring, the time between CAC and BAC imaging, and final conclusions (Table 2).

**Table 2:** Article analysis comparing BAC and CAC BAC, Breast artery calcification; CAC, Coronary artery calcification; F, Female; NPV, Negative predictive value; OR, Odds ratio

Article	Study design	Number of patients with BAC	Mean age	BAC score	Correlation of BAC with CAC score	Comparison of BAC presence with the Agatston score	Maximum time between BAC and CAC scoring	Conclusion
Chadashvili et al. [19]	Retrospective review	145 F (37 with BAC and 108 without BAC)	58.5	Not reported	BAC correlated with CAC score of >11 Agatston score (p=0.0001), indicating a mild or greater risk of developing CAD; a CAC score of >11 Agatston score was observed in 68% of BAC+ and 31% of BAC- patients.	BAC-positive group: (0 Agatston units (n=4), 1-10 Agatston units (n=3), 11-100 Agatston units (n=13), 100-400 Agatston units (n=9), >400 Agatston units (n=8)). BAC-negative group: (0 Agatston units (n=55), 1-10 Agatston units (n=30), 11-100 Agatston units (n=11), 100-400 Agatston units (n=5), >400 Agatston units (n=6))	12 months	BAC does predict CAC score of >11 Agatston score; increasing score of BAC is associated with an elevated CAC score
Yurdasik et al. [20]	Retrospective review	31 F (30 with BAC and 1 without BAC)	57	Score 0 (n=1), score 1-3 (n=9), and score 4-12 (n=21)	BAC and CAC scores were increased as the age of the patients increased; CAC score of 0 with a BAC score of 0 (n=1). CAC score of 0 with a BAC score of 1-3 (n=7), CAC score of 11-100 with a BAC score of 1-3 (n=2), CAC score of 11-100 with a BAC score of 4-12 (n=10), CAC score of 101-400 with a BAC score of 4-12 (n=8), CAC score of >400 with a BAC score of 4-12 (n=3); The correlation between BAC and CAC scores was statistically significant (r=0.796 p<0.005).	0 Agatston units (n=8), 11-100 Agatston units (n=12), 100-400 Agatston units (n=8), AND >400 Agatston units (n=3)	30 months	Positive correlation between BAC and CAC scores. This correlation between BAC and CAC scores could be used as a diagnostic tool in asymptomatic women. Majority of patients were found to have a BAC of 4-12 with a CAC 11-100 (47.6%). The correlation between BAC and CAC scores was statistically significant.
Kamel et al. [21]	Retrospective review	222 F (56 with BAC and 166 without BAC)	60	Not reported	84% (47/56) of women had both BAC and CAC; significant association between BAC and CAC scoring	Not provided	6 months	Women with BACs have a high specificity for CAC. The reporting of BACs should prompt clinicians to risk stratify women for atherosclerotic disease. These women may otherwise be undetected by conventional risk calculators.
Yoon et al. [22]	Retrospective cohort study	126 F (all with BAC)	54.5	Not reported	27/126 had BAC and CAC. Women with BAC were more likely to have CAC at both baseline (55.6% vs. 15.7%).	Not completed	51.6 months	BAC is independently associated with the progression of CAC.
Margolies et al. [23]	Retrospective cohort	292 F (124 with BAC and 168 without BAC)	61.5	Mean score=2.2	Patients with BAC were significantly older than those without BAC (66.5 years versus 57.7 years, p<0.001). Patients with CAC (n=137) were significantly older than those without CAC (n=153) (66.6 years versus 56.8 years, p<0.001). BAC and CAC scores increased with age (p<0.001). The frequency distributions of the number of calcified breast arteries, maximum length of calcification, and maximum density of the calcifications for the three age categories rose with increasing age (p<0.0001). CAC of 0 with BAC 0 (76%), BAC 1-3 (10%), and BAC 4-12 (14%). CAC of 1-3 with BAC 0 (43%), BAC 1-3 (20%), and BAC 4-12 (37%). CAC of 4-12 with BAC 0 (27%), BAC 1-3 (17%), and BAC 4-12 (56%)	Not provided	12 months	There is a strong quantitative association of BAC with CAC. BAC is superior to standard cardiovascular risk factors.
Fathala et al. [24]	Retrospective review	307 F (142 with BAC and 165 without BAC)	57	Mild BAC (n=48), moderate BAC (n=56), and severe BAC (n=38)	A strong correlation was found between BAC and total CAC (p=0.001). A strong correlation was found between CAC and the components of the BAC score (number of calcified vessels, density of the calcification, and length of the calcified vessels) (p<0.0001)	Not provided	12 months	Strong association between BAC and CAC
Yoon et al. [25]	Retrospective review	2100 F (199 with BAC and 1901 without BAC)	52	Score 0 (n=1901), score 1-6 (n=138), and score 7-12 (n=61)	BAC was an independent predictor of CAC (OR: 3.54-2.22). BAC score of 1-6 was significant associated with the presence of CAC (p<0.001, OR :1.86-4.34). BAC score of 7-12 was significant associated with the presence of CAC (p<0.001, OR: 3.18-9.61). Average score of CAC was 10.1 Agatston units. CAC of 0 with BAC score of 0 (n=1755), CAC of 0 with BAC score of 1-6 (n=108), CAC of 0 with BAC score of 7-12 (n=38), CAC present with BAC score of 0 (n=153), CAC present with BAC score of 1-6 (n=27), and CAC present with BAC score of 7-12 (n=19).	Not provided	Same day CAC and BAC testing	BAC is a significant predictor for the development of cardiovascular disease. It is an independent predictor for the development of CAC.
Deeg et al. [26]	Retrospective review	443 F (119 with BAC and 324 without BAC)	62.58	Grade 1 (n=77), grade 2 (n=33), and grade 3 (n=9)	176 out of the 324 patients without BAC did have CAC. 22 out of the 324 patients without BAC were found to have severe CAC above >300 Agatston units. When stratified by age groups (<55, 55-65, >65 years), those in <55-year-old group were significantly found to have elevated rates of BAC and CAC of 0 (p<0.001), those in the 55-65-year-old group were found to significantly have elevated rates of BAC of 0 with a CAC score of >300 Agatston units (p<0.0004), and those in the >65-year-old group were found to significantly have elevated rates of BAC of 0 with a more than 50% stenosis of their coronary vessels (p<0.0003).	Not provided	12 months	BAC 0 rules out severe CAC of >300 Agatston units; in females <55 years only; however, not those in increasing age groups, as BAC is linked to elevated CAC in older age
McLenachan et al. [27]	Randomized control trial	405 F (93 with BAC and 312 without BAC)	59	Grade 0 (n=312), grade 1 (n=30), grade 2 (n=32), and grade 3 (n=31)	BAC was associated with CAC (p=0.018). Patients with BAC had a higher median CAC score (p=0.006). Patients with BAC were more likely to have coronary artery disease (p<0.02, 62% versus 49%). Patients with BAC were more likely to have obstructive coronary artery disease with >70% stenosis (p<0.058, 20% versus 13%).	Not provided	22 months	Patients with more severe BAC were older, with a higher cardiovascular risk score, and were more likely to have a family history of coronary artery disease or be non-smokers. Patients without BAC were very unlikely to have severe CAC (NPV: 95%), but the diagnostic accuracy of BAC to identify coronary artery disease was poor.
Soylu et al. [28]	Retrospective review	404 F (123 with BAC and 281 without BAC)	61.2	Grade 1 (n=44), grade 2 (n=40), and grade 3 (n=39)	BAC-positive patients were found to more likely have CAC (45.5% versus 19.9%, p<0.001). Patients with BAC were significantly older than those without BAC (54.5 years versus 67.9 years, p<0.001). More severe calcifications (grade 2 and 3) in the BAC-positive group. Grade 1 CAC with BAC-positive (22%) versus BAC-negative (14.9%) (p<0.085), grade 2 CAC with BAC-positive (15.4%) versus BAC-negative (3.6%) (p<0.001), and grade 3 CAC with BAC-positive (8.1%) versus BAC-negative (4%) (p<0.001). BAC-positive versus BAC-negative in left main CAC: 22.8% versus 6.8% (p<0.001). BAC-positive versus BAC-negative in left descending artery calcification: 39.8% versus 17.1% (p<0.001). BAC-positive versus BAC-negative in left circumflex artery calcification: 24.4% versus 5.7% (p<0.001). BAC-positive versus BAC-negative in right CAC: 24.4% versus 5.3% (p<0.001)	Not provided	6 months	Patients with BAC were significantly older than those without BAC (54.5 years versus 67.9 years; p<0.001). CAC was found to be more common in women with BAC, and the degree of BAC was found to be associated with higher levels of CAC.
Oksul et al. [29]	Retrospective review	297 F (41 with BAC and 256 without BAC)	57.9	Grade 0 (n=256), grade 1 (n=25), grade 2 (n=9), and grade 3 (n=7)	Median CAC score was significantly higher in patients with BAC (5.5 versus 0; p = 0.001). Patients with BAC had higher moderate CAC score (21.3% versus 13.9%; p=0.001)C	BAC-positive group: (0 Agatston units (n=21), 1-10 Agatston units (n=3), 11-100 Agatston units (n=10), 100-400 Agatston units (n=9), and >400 Agatston units (n=4)). BAC-negative group: (0 Agatston units (n=171), 1-10 Agatston units (n=46), 11-100 Agatston units (n=38), 100-400 Agatston units (n=10), and >400 Agatston units (n=8))	12 months	The absence of BAC practically excludes severe coronary artery disease. The presence and severity of BAC detected in routine mammography can alert to increased cardiovascular risk without any additional imaging and radiation exposure.
Saenger et al. [30]	Retrospective cohort review	124 F (22 with BAC and 102 without BAC)	57	Grade 1 (n=83), grade 2 (n=18), grade 3 (n=13), and grade 4 (n=9)	91% in the BAC group had a CAC score compared with 46% in the non-BAC score group (p<0.01). BAC was more prevalent among the patients with CAC (p<0.001). The severity of CAC increased with the BAC score: In grade 1 BAC, 15% exhibited CAC, in grade 2 BAC, 31% exhibited CAC, in grade 3 BAC, 38% exhibited CAC, and in grade 4 BAC, 44% exhibited CAC. Those with BAC had an elevated number of coronary vessels affect (p<0.01): 55% of the BAC-negative group had zero coronary vessels impacted compared with 9% in BAC group, 22% of the BAC-negative group had one coronary vessels impacted compared with 32% in BAC group, 11% of the BAC-negative group had two coronary vessels impacted compared with 32% in BAC group, 8% of the BAC-negative group had three coronary vessels impacted compared with 18% in BAC group, and 5% of the BAC-negative group had four coronary vessels impacted compared with 9% in BAC group. The left anterior descending artery, right coronary artery, and circumflex artery were significantly more often affected in the BAC group (p<0.01, p<0.02, p<0.01, respectively); No significant difference was observed for the left main coronary artery (p=1)	BAC-positive group: (0 Agatston units (n=2), 1-10 Agatston units (n=5), 11-100 Agatston units (n=6), 100-400 Agatston units (n=4), and >400 Agatston units (n=5)). BAC-negative group: (0 Agatston units (n=56), 1-10 Agatston units (n=19), 11-100 Agatston units (n=9), 100-400 Agatston units (n=13), and >400 Agatston units (n=5))	35.3 months	Vascular calcifications in breast tissue seen via mammography correlated significantly with CAC.
Seifi et al. [31]	Cross-sectional study	60 F (50 with BAC and 10 without BAC)	49.52	Mild (n=39), moderate (n=9), and severe (n=2)	There was a significant correlation between coronary calcification and breast artery calcification (p=0.001). Significant relationship between coronary calcification and post-menopausal breast calcification (p<0.001)	BAC-positive group: (0 Agatston units (n=16), 1-10 Agatston units (n=13), 11-100 Agatston units (n=13), 100-400 Agatston units (n=6), and >400 Agatston units (n=2)); BAC-negative group: (0 Agatston units (n=7), 1-10 Agatston units (n=2), 11-100 Agatston units (n=1), 100-400 Agatston units (n=0), and >400 Agatston units (n=0))	2 months (n=7), 3 months (n=8), 4 months (n=20), 5 months (n=16), and 6 months (n=9)	BAC was found to be associated with CAC.
Bui et al. [32]	Retrospective review	293 F (79 with BAC and 214 without BAC)	62	Not mentioned	BAC scores were significantly correlated with CAC Agatston scores (r=0.38, p<0.001).	Not provided	12 months	BAC is significantly associated with CAC, especially among younger women in whom the presence of CAC may be more concerning.

The scoring systems used to analyze BAC and CAC scores varied. Table 2 displays the scoring system used by each study for BAC. The most common BAC scoring systems were designed using Margolies et al.'s 12-point scale, which analyzed three categories (number of calcified vessels, severity of calcification, and length of calcification) to develop a BAC score [23]. The other common scoring system was developed based on Mostafavi et al.'s 4-point system, which described the type of calcification within the breast arteries [33]. Other studies developed their own systems for classifying BAC, which are listed below (Table 3).

**Table 3:** Breast artery calcification scoring systems used by each study BAC, Breast artery calcification; BI-RADS, Breast Imaging-Reporting and Data System

Author	BAC scoring system used	Description of the scoring system
Chadashvili et al. [19]	No scoring system was used; they only identified whether BAC was present or absent.	-
Yurdasik et al., Kamel et al., Yoon et al., and Margolies et al. [20–23]	12 score (0=none, 1-3=mild calcification, and 4-12 severe calcification)	The score is made up of three categories: 0-6 points for the number of calcified vessels, 0-3 points for the severity of calcification, and 0-3 points for the length of calcified vessels.
Fathala et al. [24]	12 score (0=none, 1-4=mild calcification, 5-8=moderate calcification, and 9-12=severe calcification)	The score is made up of three categories: 0-6 points for the number of calcified vessels, 0-3 points for the severity of calcification, and 0-3 points for the length of calcified vessels.
Yoon et al. [25]	12 score (0=none, 1-6=mild calcification, and 7-12=severe calcification)	The score is made up of three categories: 0-6 points for the number of calcified vessels, 0-3 points for the severity of calcification, and 0-3 points for the length of calcified vessels.
Deeg et al., McLenachan et al., and Soylu et al. [26–28]	4-point scale (0=none, 1=mild, 2=moderate, and 3=severe)	Grade 0=no calcification, grade 1=few punctate vascular calcifications with no coarse, tram track, or ring calcifications, grade 2=coarse vascular calcification or tram track calcification in fewer than three vessels, and grade 3=severe coarse or tram track calcification affecting three or more vessels
Oksul et al. [29]	4-point scale (0=none, 1=mild, and 2=moderate, 3=severe)	Grade 0=no calcification is graded, grade 1=few scattered punctate or short linear calcifications, grade 2=more abundant punctate or short linear calcifications, and grade 3=continuous circumferential calcifications
Saenger et al. [30]	4-point Likert scale following BI-RADS criteria	Grade 1=no visible or sporadic scattered microcalcifications, grade 2=few discontinuous calcified particles visible in a single location and with questionable association with a tubular structure mimicking fibroglandular microcalcifications, grade 3=calcifications clearly originating from BAC but of a modest degree, and grade 4=substantial BAC
Seifi et al. [31]	3-point simplified scale	Mild = scant calcification in vessels, in which the distance between the calcifications was at least 10 cm, moderate = calcification of the complete length of an artery, and severe = complete calcification in multiple arteries
Bui et al. [32]	Did not specify	-

Table 4 displays the scoring systems used for classifying CAC scores. The most commonly used and established scoring system was the Agatston scoring system, established in the 1990s [34]. Another less common option was analyzing the luminal obstruction amount; however, the optimal cut-off as to what is classified as mild, moderate, severe, etc., varied based on the study’s predetermined limits. Also, others measured the number of calcified vessels and the length of calcification to determine their reported evaluation of the CAC score.

**Table 4:** Coronary artery calcification scoring systems used by each study

Author	CAC scoring system used	Description of the scoring system
Chadashvili et al., Yurdasik et al., Deeg et al., Saenger et al., Seifi et al., Yoon et al., McLenachan et al., Fathala et al. [19,20,24–27,30,31]	Agatston scoring system	0 Agatston units = no risk, 1–10 Agatston units = minimal, 11–100 Agatston units = mild, 101–400 Agatston units = moderate, >400 Agatston units = high risk
Deeg et al. [26]	5-point scoring based on coronary stenosis severity	Minimal = <25% obstruction, mild = 25%-49.9% obstruction, moderate = 50%-69.9% obstruction, severe = ≥70%-99% obstruction, occluded = 100% obstruction
Oksul et al. and Yoon et al. [22,29]	Obstruction scoring	Normal = no atherosclerosis, non-critical stenosis = <50% occlusion, critical stenosis = 50% occlusion
Kamel et al. [21]	Scored as present or absent	-
McLenachan et al. [27]	Obstruction scoring	Normal = <10% luminal cross-sectional area stenosis, non-obstructive = 10%–70% stenosis, obstructive = 70% obstruction in one or more major epicardial vessel or >50% in the left main stem
Soylu et al. [28]	3-point score	Grade 0 = absence of calcification, grade 1 = presence of 1-2 calcification foci, grade 2 = single calcification extending >2 foci or ≥2 sections, grade 3 = calcification along a long coronary artery segment
Margolies et al. [23]	12-point scale (grade 0 = absent, grade 1 = mild, grade 2 = moderate, grade 3 = severe) with four arteries being scored	Grade 1 (mild) = less than one-third of the length of the entire artery showed calcification, grade 2 (moderate) = one-third to two-thirds of the artery showed calcification, grade 3 (severe) = more than two-thirds of the artery showed calcification.
Bui et al. [32]	Did not specify	-

As mentioned previously, only women were included in the studies. The time between CAC and BAC scoring ranged from the same day to 2.94 years, with a mean of 16.2 months and a median of 12 months. All studies found a positive correlation between the presence of BAC and CAC, indicating that the presence of BAC may warrant further imaging of the coronary vessels, even if the women present with no other cardiovascular risk factors. Interestingly, studies additionally found that women with CAC and BAC were older, and the risk of CAC increased with age [21,23,26,28]. Furthermore, it was found that those with BAC had a greater extent of stenosis of the coronary arteries, a greater number of coronary arteries with calcification presentation, and a greater distribution of calcification along the vessels [24,27-30]. Oksul et al. were the only study to analyze mortality, and they found greater rates of cardiovascular mortality in women with the presence of BAC [29].

To further understand the association and ability to determine CAC risk based on BAC, seven studies analyzed the sensitivity, specificity, positive predictive value (PPV), and negative predictive value (NPV) (Table 5). Sensitivity was measured in five out of seven studies, with a range of 23%-63% for overall findings and an average sensitivity of 38.9%. Specificity was measured in six out of seven studies, with a range of 76%-96.7% for overall findings and an average sensitivity of 88%. PPV was measured in all studies, with a range of 27.1%-90.9% for overall findings and an average of 66.5%; however, the majority of studies indicated a PPV above 50%. With the removal of the outlier, the presence of BAC is moderately likely to identify individuals who truly also have CAC. NPV was measured in all studies, with a range of 51%-90.5% for overall findings and an average of 57.2%.

**Table 5:** Sensitivity, specificity, positive predictive value, and negative predictive value of BAC determining CAC BAC, Breast artery calcification; CAC, Calcium artery calcification; CAD, Coronary artery disease

Author	Sensitivity	Specificity	Positive predictive value	Negative predictive value
Oksul et al. [29]	Not analyzed	Not analyzed	55.3%	62.3% overall, 97.6% CAC score >400
Bui et al. [32]	Not analyzed	88% overall, 95% <60 years old, 80% 60-69 years old, and 88% >70 years old	77% overall, 75% <60 years old, 66% 60-69 years old, and 88% >70 years old	Not analyzed
Saenger et al. [30]	30.3%	96.7%	90.9%	54.9%
Kamel et al. [21]	51%	93%	84%	72%
Yoon et al. [25]	23% overall and 9.36% with a BAC>6	92.2% overall and 98% with a BAC>6	27.1% overall and 36.7% with a BAC>6	90.5% overall and 63.3% with a BAC>6
McLenachan et al. [27]	27% overall, 33% severe CAC, 28% any CAD, and 33% obstructive CAD (>70% stenosis)	82% overall, 78% severe CAC, 82% any CAD, and 79% obstructive CAD (>70% stenosis)	62% overall, 9% severe CAC, 62% any CAD, and 20% obstructive CAD (>70% stenosis)	51% overall, 95% severe CAC, 51% any CAD, and 88% obstructive CAD (>70% stenosis)
Margolies et al. [23]	63%	76%	69%	70%

Discussion

CVD is the leading cause of death among American women; however, there is a greater worry among women regarding identifying the presence of breast cancer rather than cardiovascular conditions [35]. Women, on average, complete regular mammograms starting at the age of 40 years as per the American College of Radiology recommendations due to the worry of breast cancer, but do not complete regular CVD screening. As BAC is often an incidental finding identified on mammograms, recent evidence suggests that it has potential usefulness as a primary indicator for patients to undergo further cardiovascular testing.

The link between BAC and CVD has been a well-explored topic. In a meta-analysis analyzing over 87,000 patients, BAC was significantly associated with an elevated risk of developing CVD, including hemorrhagic stroke (p=0.003), ischemic stroke (p<0.00001), peripheral vascular disease (p=0.003), and heart failure (p<0.00001) [11]. It has also been noted that those with BAC have a higher associated all-cause and cardiovascular mortality independent of traditional risk factors and have a significantly increased risk of cardiovascular negative outcomes, including heart failure (p<0.001), myocardial infarction (p<0.001), and stroke (p<0.001) [36,37]. Women with BAC were found to be at a 23% increased risk of the development of any form of CVD [9]. Due to these findings, there has been a greater push to report the presence and extent of BAC to patients and their primary care providers to complete further testing. This review, in particular, noted a significant association between the presence of BAC and the development of CAC.

Recent studies have come out regarding the risk of CVD and the presence of both BAC and CAC. It was recently found that those with both present were at an elevated 10-year CVD risk (13.3%) compared with those with only BAC (8.8%) or only CAC present (5.8%) [38]. As a result, this study aimed to better understand the relationship between BAC and CAC and whether BAC could be used as an indicator of CAC. All studies analyzed within this review identified a positive relationship between the presence of BAC and CAC. Specifically, it was also noted that those with higher BAC scores could also be associated with having higher CAC scores as well [20,23,25,28-30,32]. Furthermore, for the studies that analyzed CAC using the gold standard scale of Agatston units, it was found that the presence of BAC was associated with a greater number of patients found with moderate-to-severe stratification of CAC compared with those without BAC [19,20,29-31]. Some studies reviewed also analyzed stenosis rates and found that with the presence of BAC, there was greater stenosis of coronary vessels [26,27,29]. Particularly, two studies analyzed the association between BAC presence and the number and severity of coronary arteries affected. Saenger et al. found that those with BAC had an elevated number of coronary arteries affected, and the left anterior descending artery, right coronary artery, and left circumflex artery were more commonly affected, but no change in the left main coronary artery was observed [30]. Solyu et al. found similar findings; however, they concluded that all vessels had greater occlusion, including the left main coronary artery [28].

The pathophysiological relationship between BAC and CAC requires much further analysis. However, preliminary research currently points to the activation of vascular smooth muscle cells being the link between these two conditions. Stress on vascular smooth muscles has been found to trigger multiple processes, increasing calcification deposits within the vascular walls of both coronary and breast arteries. Hypotheses include the increased release of calcifying proteins such as glucose-regulated protein 78, which influences the expression of osteogenic markers of Runx2, osteoprotegerin, alkaline phosphatase, osterix, and bone sialoprotein, which all increase calcium deposition [39]. Additionally, the lack of expression of calcification inhibitors such as Gla protein can lead to unregulated expression of matrix metalloproteinase-2 activity, which triggers the expression of bone morphogenetic protein-2, runx2, and msh homeobox 2, driving the calcification of vascular smooth muscle [40]. Further research is required in this area to better understand whether a pathophysiological correlation exists between the calcification of coronary and breast arteries, as calcification often arises in different layers of the vessels: intimal for coronary and medial for breast.

It is also important to assess the BAC's potential to be used as a screening test. When analyzing the sensitivity, specificity, PPV, and NPV reported in seven of the studies, it was found that BAC has a low sensitivity, high specificity, moderate PPV, and low NPV. Due to BAC indicating a high specificity and low sensitivity, it has a high probability of ruling in disease.

An interesting reported finding was the association between BAC and CAC presence in older age groups [21,23,26,28,31]. Women in older age groups were associated with a higher prevalence, and there was an association between BAC and CAC. This association may be due to the significant decline in estrogen levels, which is protective against vascular calcifications. Traditionally, this association was connected to the protection of coronary vessels; however, the protective elements may also extend to breast arteries. Estrogen inhibits the osteogenic differentiation of vascular smooth muscle cells [41]. Recent evidence has pointed to hormone replacement therapy (HRT) reducing the prevalence of both BAC and CAC. Studies have found that, among women aged above 65 years, those who have used HRT had a significantly lower prevalence of BAC (25.8%) compared with those who did not use HRT (74.2%) [42]. Similarly, women who used HRT had a significantly lower prevalence of CAC, with a 50% reduction in CAC levels and lower calcium levels in the coronary arteries [43,44]. A potential recommendation based on these findings is to introduce HRT for all post-menopausal women, which, in essence, may reduce subsequent cardiovascular risk factors and calcification of both the coronary and breast arteries.

Due to mammograms being the most widely used screening test in women, with approximately 78.7% completing their mammograms on schedule, and with their ability to detect BAC in addition to monitoring for signs of cancer, it potentially could be a screening tool for CVD and CAC without the need for additional testing or radiation exposure [31]. Additionally, it could be particularly beneficial as an additional CVD assessment tool in women who lack access to primary care physicians due to finances, transportation, social isolation, etc. It has been found that there has been a lack of completion of the 10-year CVD score for everyone above the age of 40 years, as only 54% of general practitioners regularly perform this screening [45]. Potentially, the identification of BAC within mammograms could enhance the completion of the 10-year CVD screening test. It is important to note that due to low sensitivity and NPV, the absence of BAC does not necessarily rule out CAC. As a result, those with no BAC should still be highly recommended to complete the usual screening for CVD. BAC should be used as a potential screening tool to identify potentially healthy women with CVD but not to rule them out absolutely. A recommendation is to create a guideline for any woman with BAC in their mammogram to be referred for a CT angiogram to complete a CAC, as well as referred to a cardiologist or their family physician for an in-depth cardiovascular risk analysis. Furthermore, it is crucial to note that healthy women with no underlying concerns will not receive their first mammogram until around 40 years of age. As a result, there is limited data on the detection of BAC in younger women. Further research should be completed in younger age groups as well to see if the correlation between BAC and CAC exists. The identification of BAC in younger women with low-CVD risk scores should still undergo further risk assessment, and the presence of BAC in itself should be counted as a risk.

BAC is a promising initial indicator of the potential of CAC; however, there is no standardized method to measure and report it. Most studies analyzed within this review used the method of indicating its presence or absence, describing the appearance of the calcification, and assigning it a grade score or indicating it as mild, moderate, or severe. The majority did follow some form of the Breast Imaging-Reporting and Data System method for categorizing the BAC based on the number of vessels calcified, the length of calcification, and the density of calcification, and providing a score of 0-12, categorized as 0 no risk, 1-6 mild risk, and 6-12 high risk. However, to our knowledge, no method has yet been developed to quantify the amount of calcification within the breast arteries, such as the Agatston units used in CAC. Moving forward, further research should be conducted to develop a method for quantitatively measuring BAC, which could further assist in better understanding the amount of BAC that indicates low, intermediate, and high risk of CAC and CVD, and when patients should undergo further testing and imaging.

Finally, guidelines should be developed that require the reporting of BAC for all mammograms. A survey by the American College of Radiology noted that 87% of radiologists have reported BAC, but less than half (41%) state they do this always or most of the time [46]. Around 95.8% of women state that they would like to be informed of the presence of BAC [47]. Down the pipeline, if consistent BAC reporting is completed, clinical algorithms to guide the evaluation and management of patients with BAC should be established. These could better assist the judgment of primary care physicians in referring patients for additional testing, such as CAC testing and CT angiograms, and focus more attention on the lipid profile and additional markers, including high-sensitivity C-reactive protein and lipoprotein(a).

Limitations of this paper include the lack of ethnicity reporting in the studies reviewed. Prior research has shown that BAC is more prevalent among Hispanic populations, followed by Caucasians, African Americans, and Asians. Including patient ethnicity in larger-scale studies would offer valuable insights into whether certain populations are at a higher risk for both BAC and CAC, enabling more targeted and effective preventive strategies. Another limitation is the lack of analysis involving younger populations. All studies focused exclusively on post-menopausal women. While it is well-established that risk factors such as estrogen loss contribute to arterial calcification and CVD, investigating the relationship between BAC and CAC in younger women may expand the potential of BAC as a screening tool. Notably, the presence of BAC in younger women, even in the absence of CAC, could underscore the need for earlier screening and intervention to prevent adverse cardiovascular outcomes. Lastly, inconsistency in the scoring methods for BAC and CAC across studies presented challenges in comparing findings. The lack of standardized scoring systems made it difficult to draw clear conclusions about the relationship between the two types of calcifications.

## Conclusions

With millions of women undergoing mammography each year, the incorporation of BAC as a cardiovascular screening tool is an appealing strategy to enhance the clinical utility of an already widely used imaging modality. Given its accessibility, cost-effectiveness, and non-invasive nature, BAC holds strong potential as an early marker of subclinical atherosclerosis, especially in populations not routinely evaluated for CVD. The high specificity of BAC may allow it to serve as a useful tool for ruling out CAC in its absence, while any presence of BAC on mammography should prompt further cardiovascular risk assessment. Although additional research is necessary to standardize BAC scoring and validate its predictive value across diverse populations and age groups, integrating BAC evaluation into routine mammographic interpretation could significantly advance the early detection and prevention of CVD in women.
